# Treatment of *Mycobacterium abscessus* Infection

**DOI:** 10.3201/eid2203.150828

**Published:** 2016-03

**Authors:** Shannon A. Novosad, Susan E. Beekmann, Philip M. Polgreen, Kate Mackey, Kevin L. Winthrop, M. abscessus Study Team

**Affiliations:** Oregon Health and Science University, Portland, Oregon, USA (S.A. Novosad, K. Mackey, K.L. Winthrop);; University of Iowa Carver College of Medicine, Iowa City, Iowa, USA (S.E. Beekmann, P.M. Polgreen)

**Keywords:** Nontuberculous mycobacteria, adverse effects, therapeutics, treatment, *Mycobacterium abscessus*, bacteria, tuberculosis and other mycobacteria

## Abstract

*Mycobacterium abscessus* is often resistant to multiple antimicrobial drugs, and data supporting effective drugs or dosing regimens are limited. To better identify treatment approaches and associated toxicities, we collected a series of case reports from the Emerging Infections Network. Side effects were common and often led to changing or discontinuing therapy.

*Mycobacterium abscessus* infections are challenging to treat because multidrug resistance necessitates prolonged intravenous (IV) therapy and side effects are perceived to be common. For the best chance of pulmonary disease cure, guidelines from the American Thoracic Society/Infectious Diseases Society of America (ATS/IDSA) recommend multidrug macrolide-based therapy based on susceptibility testing results and surgical resection. However, these guidelines also state that there are no drug combinations with proven efficacy (*1*). Similarly for extrapulmonary disease, macrolide-based treatment regimens based on susceptibility testing results are recommended (*1*,*2*). Inducible macrolide resistance in many strains of *M. abscessus* further complicates treatment (*3*). Given the lack of evidence-based therapies, we hypothesized that treatment regimens have no clear pattern and that medication changes and toxicities occur frequently.

## The Study

The Emerging Infections Network (EIN) gathers information about emerging infectious diseases in North America (*4*) and is frequently used for case collection. The EIN is funded through a cooperative agreement between the Centers for Disease Control and Prevention and IDSA. To learn more about treatment regimens and associated side effects, during March–December 2013, we asked EIN physician members to report recent cases of *M. abscessus* via an emailed electronic data collection form.

A total of 65 cases were reported from 16 states; patient mean age was 53.6 years. Most cases were in white, nonsmoking women. Concurrent conditions included cystic fibrosis (n = 9, 14%), cancer (n = 7, 11%), and chronic obstructive pulmonary disease (n = 6, 9%). Ten (15%) patients had used immunosuppressive medications in the 3 months before diagnosis. Most (36 [55%]) organisms were reported as *M. abscessus* complex, 27 (42%) as *M. abscessus*, and 2 (3%) as *M. massliense*. According to available records, at the time of case report, 55 (85%) patients had started or finished antimicrobial drug therapy.

Of the 65 patients, 41 (63%) had pulmonary *M. abscessus* infection; 19 isolates were from bronchoalveolar lavage fluid and 16 from >2 sputum samples. Of these 41 patients, 34 (83%) started antimicrobial drug therapy. Among those not starting therapy, 2 opted for monitoring only, 1 died before therapy was started, and 4 had no reason reported. A total of 21 initial medication combinations were reported (Table 1). The most commonly reported medications were IV amikacin (n = 22, 65%) and azithromycin (n = 24, 71%). The most commonly used regimen was IV amikacin, a second IV agent, and a macrolide (n = 15, 44%). Only 5 patients received no IV agents. Twenty-eight (82%) patients required a change in therapy (because of side effects, lack of effectiveness, or need for suppressive regimen); 3 underwent surgical therapy, and 12 stopped therapy (median duration 12 months, interquartile range [IQR] 9–18 months).

**Table 1:** Initial drug regimens for pulmonary and extrapulmonary *Mycobacterium abscessus* infection and therapy-modifying/ending side effects*

Regimen, no. patients	Total no. (%) patients	Therapy-modifying/ending side effect, no. (%) patients
Pulmonary disease, 33†		
Non-IV agents	5 (15)	2 (40)
Clarithromycin, linezolid, 1		
Azithromycin, inhaled amikacin, ethambutol, rifampin, 2		
Azithromycin, inhaled amikacin, ethambutol, moxifloxacin, 1		
Azithromycin, ethambutol, linezolid, 1		
Single IV agent	3 (9)	1 (33)
Amikacin, azithromycin, levofloxacin, 1		
Tigecycline, inhaled amikacin, clofazimine, 1		
Cefoxitin, azithromycin, inhaled amikacin, 1		
Dual IV agents	24 (73)	15 (63)
Amikacin/macrolide-based regimens	19 (57)	13 (68)
Amikacin, macrolide, and 1 IV agent, in addition to amikacin	15 (45)	10 (67)
Amikacin, azithromycin, tigecycline, 7		
Amikacin, clarithromycin, cefoxitin, 2		
Amikacin, azithromycin, cefoxitin, 4		
Amikacin, azithromycin, imipenem, 2		
Amikacin, macrolide, 1 IV agent in addition to amikacin, and other oral agents	4 (12)	3 (75)
Amikacin, azithromycin, cefoxitin, moxifloxacin, 1		
Amikacin, clarithromycin, cefoxitin, moxifloxacin, 1		
Amikacin, azithromycin, imipenem, ethambutol, rifampin, 1		
Amikacin, clarithromycin, cefoxitin, other, 1		
Other amikacin-based regimens	1 (3)	0
Amikacin, cefoxitin, 1		
Regimens without IV amikacin	4 (12)	2 (50)
Azithromycin, imipenem, tigecycline, 1		
Clarithromycin, tigecycline, imipenem, 1		
Clarithromycin, moxifloxacin, tobramycin, cefoxitin, 1		
Azithromycin, inhaled amikacin, cefoxitin, imipenem, 1		
Triple IV agents	1 (3)	1 (100)
Amikacin, macrolide, and 2 IV agent, in addition to amikacin	1 (3)	1 (100)
Amikacin, azithromycin, tigecycline, cefoxitin, 1		
Extrapulmonary disease, 21		
No IV agents	9 (43)	1 (11)
Clarithromycin, other, 1		
Clarithromycin, doxycycline, 1		
Moxifloxacin, tobramycin drops, azithromycin drops, other, 1		
Azithromycin, linezolid, 1		
Tobramycin drops, azithromycin, moxifloxacin drops, azithromycin topical, 1		
Levofloxacin, doxycycline, 1		
Ciprofloxacin, minocycline, 1		
Clarithromycin, minocycline, 1		
Azithromycin, moxifloxacin, 1		
Single IV agent	5 (24)	2 (40)
Amikacin, azithromycin, clofazimine, 1		
Cefoxitin, azithromycin, 1		
Amikacin, ethambutol, 1		
Imipenem, azithromycin, moxifloxacin, 1		
Imipenem, azithromycin, ciprofloxacin, 1		
Dual IV agents	6 (29)	5 (83)
Amikacin-based regimens	6 (29)	5 (83)
Amikacin, macrolide and one IV agent, in addition to amikacin	5 (24)	4 (80)
Amikacin, azithromycin, imipenem, 2		
Amikacin, clarithromycin, cefoxitin, 1		
Amikacin, clarithromycin, imipenem, 2		
Amikacin, macrolide,1 IV agent, in addition to amikacin, and other oral agents	1 (5)	1 (100)
Amikacin, clarithromycin, cefoxitin, moxifloxacin, linezolid, 1		
Triple IV agents	1 (5)	1 (100)
Amikacin, macrolide, 2 IV agents, in addition to amikacin, and oral agent	1 (5)	1 (100)
Amikacin, clarithromycin, imipenem, tigecycline, clofazimine, 1		

*IV, intravenous. †Therapy was started for 34 patients, but 1 initial regimen was unknown.

Of the 24 patients with extrapulmonary disease (median age 50 years, IQR 42–66 years), most (17 [71%]) had skin or soft tissue infections. Also reported were 2 corneal, 1 peritoneal, 1 catheter-related, and 1 pacemaker pocket infection plus 1 case each of endocarditis and osteomyelitis. Medical therapy had been started by 21 (88%) patients. Reasons for not starting therapy included being lost to follow-up, declining therapy, or being referred for surgery without antimicrobial drugs. The most commonly used agents were IV amikacin (n = 9, 43%), macrolides (n = 18, 86%), and imipenem (n = 7, 33%) (Table 1). Regimens that contained >1 IV agent were administered to 12 (57%) patients; IV amikacin–based regimens with a macrolide and 1 other IV agent were administered to 5 (24%). Change from the initial therapeutic regimen was needed by 14 (67%) patients. Among the 15 patients who stopped therapy, median duration of therapy was 6 months (IQR 4–8 months); 14 (93%) stopped therapy because of improvement or presumed cure. Fourteen (58%) of the 24 patients with extrapulmonary disease underwent surgery.

Side effects were common; 74 side effects were documented among 34 (62%) of 55 patients who received treatment. Most common were nausea/vomiting (n = 17, 31%) and skin changes (n = 11, 20%) (Table 2). When the specific medication causing a side effect was known, it was most commonly amikacin (22 [30%]) or tigecycline (13 [18%]). Of the 9 reported episodes of renal insufficiency, 7 were attributed to amikacin. IV agents were commonly associated with side effects that often required dosage adjustment or discontinuation. Among those receiving amikacin and tigecycline, 51% and 36% of patients, respectively, had to adjust or stop medication because of side effects. Intermittent dosing of amikacin seemed to cause fewer side effects than daily dosing (42% vs. 77%, respectively). Among patients with renal insufficiency attributed to amikacin, 71% were receiving it daily.

**Table 2:** Side effects associated with antimicrobial drug therapy for *Mycoobacterium abscessus* infection, March–December 2013*

Side effect	No. (%)
Gastrointestinal†	23 (41.8)
Skin changes‡	11 (20.0)
Renal insufficiency	9 (16.4)
Hearing loss	7 (12.7)
Tinnitus	6 (10.9)
Loss of balance	4 (7.3)
Transaminitis	4 (7.3)
Shortness of breath or airway irritation	3 (5.5)
Neutropenia or thrombocytopenia	2 (3.6)
Other§	5 (9.1)

*Data for 55 patients; some patients reported the same adverse event >1 time and attributed it to different medications. Here, each adverse event is reported only 1 time. †Nausea/vomiting, abdominal pain, diarrhea. ‡Rash, pruritis, discoloration. §Anxiety, failure to thrive, fatigue, oral and genitourinary candidiasis.

Among patients with pulmonary infection, antimicrobial drug therapy was completely discontinued for 4 because of side effects. No patients with extrapulmonary disease completely stopped therapy because of side effects. Overall, >54 medication changes among 30 patients were made because of side effects or intolerance.

At the time of data collection, 8 patients had died: 6 with pulmonary and 2 with extrapulmonary disease. Of these 8 patients, 6 died while receiving therapy (5 pulmonary, 1 extrapulmonary).

## Conclusions

Our series showed a wide range of treatment strategies for *M. abscessus* infection; most consisted of prolonged antimicrobial drug therapy. Side effects were common, and therapy often needed to be changed or stopped. Amikacin, the most commonly used IV agent, was associated with multiple side effects; amikacin therapy was stopped or adjusted for 51% of patients.

Heterogeneity of initial treatment regimens was less among those with pulmonary disease than among those with extrapulmonary disease, but regimens still varied widely. However, despite the guidelines, surgical therapy was uncommon for patients with pulmonary disease; only 3 patients in this series underwent surgery.

In a retrospective analysis of 41 patients with *M. abscessus* pulmonary disease in South Korea, 18 (43.9%) patients experienced side effects (*5*). This percentage is lower than what we found (62%), possibly because a large percentage of patients in our series received amikacin or a regimen with >1 IV agent. In our series, tigecycline was used, but often as a secondary agent. A recent study of 52 patients who received tigecycline-containing salvage regimens reported improvement in 60% of patients but side effects (most commonly nausea/vomiting) in 94%; 23% of side effects were directly associated with tigecycline (*6*). Side effects from tigecycline were also common among patients in our series.

Our study had several limitations, including unknown specific subspecies of *M. abscessus*. Most isolates were reported as *M. abscessus* complex (55%) or *M. abscessus* (42%), and it is unclear if these were ever correctly identified to the subspecies level (such as *M. abscessus abscessus*). Given increasing evidence regarding varying antimicrobial drug susceptibility patterns of different subspecies, knowing if treatment patterns or side effect profiles differed between subspecies would be helpful. Incomplete information regarding duration of therapy with specific agents limited our ability to report information such as median time to any side effect or a side effect severe enough to require therapy alteration for individual medications. Although we did collect information regarding outcomes, this study was not powered to evaluate outcomes associated with individual regimens or medications. Because only EIN members could submit cases, selection bias is possible. Their treatment practices may differ from those of non-EIN members if members follow ATS/IDSA guidelines more closely.

Our survey revealed that therapeutic regimens for *M. abscessus* infection vary widely. Side effects are common and often lead to changing or discontinuing therapy. Given these findings and increasing rates of nontuberculous mycobacterial infections (*7*,*8*), prospective studies requiring cooperation across multiple centers are needed to better define appropriate treatment regimens that will maximize effectiveness while minimizing side effects.
